# Morbidity among Adolescent Hypnotic Drug Users in Norway: An Observational Population-Based Study

**DOI:** 10.3390/jcm13041075

**Published:** 2024-02-14

**Authors:** Mohammad Nouri Sharikabad, Svetlana Skurtveit, Hilchen Thode Sommerschild, Kristine Olsen, Ingeborg Hartz, Rikke Wesselhoeft, Vidar Hjellvik, Lars Johan Hauge, Marte Handal

**Affiliations:** 1 Department of Drug Statistics, Norwegian Institute of Public Health, 0213 Oslo, Norway; kristine.olsen@fhi.no; 2 Department of Chronic Diseases, Norwegian Institute of Public Health, 0213 Oslo, Norway; svetlanaondrasova.skurtveit@fhi.no (S.S.); ingeborg.hartz@fhi.no (I.H.); vidar.hjellvik@fhi.no (V.H.); marte.handal@fhi.no (M.H.); 3 Norwegian Board of Health Supervision, 0213 Oslo, Norway; hts@helsetilsynet.no; 4 Department of Research, Innlandet Hospital Trust, 2380 Brumunddal, Norway; 5 Child and Adolescent Mental Health Southern Denmark, Mental Health Services in the Region of Southern Denmark, 5000 Odense C, Denmark; rwesselhoeft@health.sdu.dk; 6 Department of Mental Health and Suicide, Norwegian Institute of Public Health, 0213 Oslo, Norway; lars.johan.hauge@fhi.no

**Keywords:** hypnotics and sedatives, melatonin, adolescent, sleep disorders, sleep disturbances, mental disorders, neurodevelopmental disorders

## Abstract

We have previously shown that the use of hypnotic drugs increased among young Scandinavians during 2012–2018. This study aimed to explore psychiatric and somatic morbidity among adolescent hypnotic drug users in a cohort study of 13–17-year-old individuals during 2008–2018 in Norway. Data sources were (i) prescription data from the Norwegian Prescription Database linked to specialist health care diagnoses from the Norwegian Patient Registry and (ii) sleep disorder diagnoses from the Primary Health Care Database. Hypnotic drugs were defined as the sedative antihistamine alimemazine and the ATC group “Hypnotics and Sedatives” (N05C), excluding midazolam. In 2017, 2519 girls (16.5/1000) and 1718 boys (10.7/1000) were incident (new) users of hypnotic drugs. Most of these new users (82% of girls, 77% of boys) were referred to secondary health care, where the most frequent diagnoses were mental and behavioral disorders (51.8% of girls, 46.2% of boys), while only 3.2% received a specific sleep disorder diagnosis. The most common mental and behavioral disorders were “Neurotic stress-related disorders” among girls (27.4%) and “Behavioral and emotional disorders” among boys (23.6%). In conclusion, the trend of increasing hypnotic drug use among adolescents reflects the initiation of hypnotic drugs in a subgroup of the population with a higher disease burden, mainly due to psychiatric disorders, than the general population.

## 1. Introduction

There is a lack of systematic monitoring of psychotropic drug use among children and adolescents in Europe and North America, with heterogeneous reporting across studies, according to a meta-analysis from 2019 [1]. We have previously shown that the use of melatonin and sedative antihistamines increased among young Scandinavians during 2012–2018 [2]. Outside Scandinavia, there was a decrease in hypnotic and sedative use in Germany from 2004 to 2012, while Icelandic data showed an increase during 2003–2007 [3,4].

In Norway, there was an increase in hypnotic drug use among adolescents until 2014 [5]. Melatonin, phenothiazines (mainly alimemazine), nitrazepam, and z-hypnotics have all been used to treat adolescents in Norway [6]. Melatonin accounted for most of the total increase in hypnotic drug use among children up to 2011. 

Until 2018, the only approved indication of melatonin in Norway was primary insomnia in patients over 55 years of age. As a result, there was a widespread and increasing off-label use of melatonin in children and adolescents in Norway [5,7] and other Scandinavian countries [2,8,9]. Treatment of sleeping problems related to underlying neurodevelopmental disorders with melatonin has been shown to be effective and has recently resulted in the approval of melatonin to treat insomnia associated with autism spectrum disorder (ASD) and/or Smith–Magenis syndrome and attention deficit hyperactivity disorder (ADHD) in children in whom sleep hygiene measures have been insufficient. In 2019, melatonin up to 1 mg became available without prescription in Norway [10].

To date, three studies have been published on diagnosed disorders among melatonin users. A study of Norwegian children who had used melatonin recurrently over a three-year period showed that approximately 90% had a psychiatric or neurological disorder [7]. A Swedish report revealed that around 50% of all young melatonin users were diagnosed with at least one psychiatric diagnosis during the previous 6 months, most commonly an ADHD diagnosis [9]. A recent Danish study focusing on melatonin use among patients aged 0–24 years showed that a substantial proportion of melatonin users had concurrent psychopathology, which most likely explained their use of melatonin [11]. Further knowledge about comorbid disorders and disease burden among adolescent users of all hypnotic drugs is needed from different populations to discuss and evaluate the observed increasing trends in use.

Along with increasing hypnotic drug use, an increase in sleep onset difficulties and a reduction in sleep duration among adolescents have also been observed [12,13]. Furthermore, an increase in self-reported symptoms of depression and anxiety among Norwegian adolescents has been observed over the last decade, especially among girls [14,15]. Sleep problems are common in depression and anxiety, and in other psychiatric and neurodevelopmental disorders such as ADHD and autism [16,17]. 

The main aim of this pharmacoepidemiologic study was to examine diagnosed psychiatric and somatic disorders among 13–17-year-old Norwegian hypnotic drug users. First, we compared the prevalence of hypnotic drug use with the prevalence of diagnosed sleep disorders in primary and secondary health care from 2008 to 2018. To capture all adolescent melatonin users before the change in legislation that made melatonin available without prescription was implemented, we identified incident hypnotic drug users in 2017 and studied any contact and diagnoses from secondary health care around the time of the hypnotic drug use and earlier during their childhood.

## 2. Materials and Methods

### 2.1. Data Sources

This study was based on data from three nationwide registries, i.e., the Norwegian Prescription Database (NorPD), the Norwegian Patient Registry (NPR), and the Norway Control and Payment of Health Reimbursements (KUHR) Database, which is part of the Primary Health Care Database. Individual-level registry data from the NPR and the NorPD were linked using the unique (encrypted) personal identity number assigned to all individuals living in Norway. Individual-level data on consultations in primary health care were available from KUHR but could not be linked to data from the NorPD and the NPR in this project. 

#### 2.1.1. The Norwegian Prescription Database (NorPD)—Information about Prescribed Hypnotic Drugs

Data on dispensed hypnotic drugs were drawn from the NorPD, which covers the entire Norwegian population (approximately 5.3 million inhabitants in 2017) [18]. Since January 2004, all Norwegian pharmacies have been obliged to send data electronically to the NorPD for all prescribed drugs dispensed to individuals in ambulatory care. Drugs administered to patients while in hospitals or institutions are not reported to the NorPD. 

In the present study, we included patients’ unique (encrypted) identity number, gender, age, the date the drug was dispensed, and drug information (based on the Anatomical Therapeutic Chemical (ATC) code and defined daily doses (DDDs)) [19]. A DDD is the assumed average maintenance dose per day for a drug used for its main indication in adults. Hypnotic drugs were defined as all drugs dispensed in Norway in ATC group N05C “Hypnotics and sedatives”, except for midazolam (N05CD08), which is mainly prescribed as oral syringes for epileptic seizures. The sedative antihistamine alimemazine (R06AD01), which is approved for insomnia in children and adolescents and has traditionally been used in Norway as a hypnotic drug for children, was also included. The different hypnotic drugs and their DDDs included in this study are shown in Table S1. All the hypnotic drugs included in this study, including melatonin, were only available on prescription in Norway during the study period. A hypnotic drug user was defined as a person who was dispensed at least one prescription of a hypnotic drug as recorded in the NorPD.

#### 2.1.2. The Norwegian Patient Registry (NPR)—Information on Diagnosis from Secondary Health Care

The NPR is an administrative database of records reported by secondary health care, i.e., all government-funded specialized hospitals and outpatient services, including private child and adolescent mental health care [20]. In Norway, there are publicly funded mental health care facilities for in- and outpatient treatment throughout the country, including clinics for child and adolescent mental health. Mental health care is free of charge for children and adolescents up to the age of 18. The private sector plays a negligible part in the health care system for children and adolescents with mental disorders. The NPR includes information on patients who have been referred by a general practitioner (GP) because of the need for secondary health care. All registered contact with secondary health care is included in the NPR. The NPR contains nationwide individual-level secondary health care data with diagnoses recorded from 2008 onwards. In this study, we used data reported by in- and outpatient clinics and substance abuse treatment facilities in the period 2008–2018. Diagnoses in the NPR are coded according to the International Classification of Diseases, 10th revision (ICD-10). Diagnostic data were obtained for ICD-10 diagnosis blocks A–Z (Table S2) and presented on a 3-digit level (e.g., F30) for selected (groups of) diagnoses in the most frequently used blocks (F, R, and Z), and on a 1-digit level for the remaining [21].

#### 2.1.3. Norway Control and Payment of Health Reimbursements (KUHR) Database—Information on Diagnosis from Primary Care

The KUHR is a Norwegian database for the control and reimbursement of health expenses in primary health care and contains data about claims for fee-for-service from 2006 and onwards [22]. For each patient-related contact in primary health care, a claim for reimbursement is sent to the Norwegian Health Economics Administration. Data from face-to-face consultations with general practitioners in the period 2008–2018 were included in this study. All claims contain an encrypted patient identifier, the date of consultation, and one or more diagnoses according to the International Classification of Primary Care second version (ICPC-2). 

### 2.2. Study Population

The study population included all adolescents aged 13–17 years in Norway in the period from 2008 to 2018. The age of hypnotic drug users was defined as the year that the hypnotic prescription was filled minus the birth year.

### 2.3. Analytical Approach

#### 2.3.1. Annual Prevalence of Hypnotic Drug Use

The annual prevalence of hypnotic drug use was calculated as the number of adolescents who were dispensed at least one prescription of a hypnotic drug in the relevant year divided by the total number of adolescents of the same age per 1 July of the same year. 

#### 2.3.2. Annual Prevalence of Sleep Disorder Diagnosis in Primary and Secondary Health Care

The annual prevalence of sleep disorders was calculated as the number of adolescents with relevant diagnosis codes in primary and secondary care, respectively, in the relevant year divided by the total number of adolescents in the same age group per 1 July of the same year. The diagnosis codes used were ICPC-2 P06, “Sleep disturbance”, in primary health care (KUHR database) and ICD-10 G47, “Sleep disorder”, and/or F51, “Sleep disorders not due to a substance or known psychological condition” in secondary health care (NPR database).

#### 2.3.3. Incident Hypnotic Drug Users

Incident (new) users were defined as adolescents who were dispensed their first hypnotic drug in 2017, as recorded in the NorPD, without having filled any prescription for a hypnotic drug during the previous two years (730 days). We studied four aspects of incident hypnotic drug use:

(a) Incidence rate

The incidence rate of hypnotic drug use in 2017 was calculated as the number of incident users in 2017 divided by the number of inhabitants in Norway in the age group 13–17 years per 1 July 2017, who were at risk of being an incident user. Incidence was studied for the total adolescent population as well as by age.

(b) Type of the first hypnotic drug use and amount dispensed to incident hypnotic drug users

The type of the first hypnotic drug filled in 2017 and the total amount of DDDs dispensed during a 365-day period after and including the first filled prescription were described in incident hypnotic drug users.

(c) Contact with secondary health care

We identified the proportion of incident hypnotic drug users who had at least one contact with secondary health care during the period from six months prior to six months after the first prescription, irrespective of diagnosis. The results were compared to the proportions of the general population of 13–17 years old in 2017 who had been in contact with secondary health care in 2017.

(d) Diagnoses from secondary health care

ICD-10 diagnoses assigned to incident users in secondary health care during the period from six months prior to six months after the first hypnotic drug prescription were identified in the NPR. We calculated the proportions of incident users who received diagnoses within the different ICD-10 blocks (Table S2). First, we studied all ICD-10 diagnoses on the A–Z block level. Then, within the most frequent A–Z blocks (for instance, F), we performed more detailed analyses of the most frequent diagnosis subblocks (for instance F30–F39).

(e) Diagnoses from secondary health care during childhood

Finally, we retrieved information on all ICD-10 diagnoses recorded before the age of 12 years among incident hypnotic drug users. The proportions of incident users with diagnoses distributed on the A–Z block level were calculated. The results were compared to the proportions among all adolescents 13–17 years in 2017 who had received a diagnosis during the same age span in childhood.

All analyses were performed using SPSS 22.0 (IBM SPSS Statistics 22, IBM Corporation, New York, NY, USA) for Windows. To assess the significance of the observed differences between the boys and girls with their respective general populations, a chi-square analysis was conducted to compare their respective statistical distributions, with a significance level set at *p* < 0.05.

### 2.4. Ethical Considerations 

This register study was approved by the Regional Committee for Medical Research Ethics (2010/131) and by the Norwegian Data Protection Authority (10/00447-5). The unique personal identity numbers used for data linkage were encrypted, resulting in a dataset that was only indirectly identifiable. Additional data minimization was performed to further reduce the possibility of indirect identification of individuals. According to privacy protection regulations, any cells with fewer than 5 participants are shown as <5. If this was not sufficient, other cells were anonymized. The datasets generated and analyzed during the current study are not publicly available due to the restrictions imposed by Norwegian legislation for privacy protection.

## 3. Results

### 3.1. Annual Prevalence of Hypnotic Drug Use

**Figure 1:**
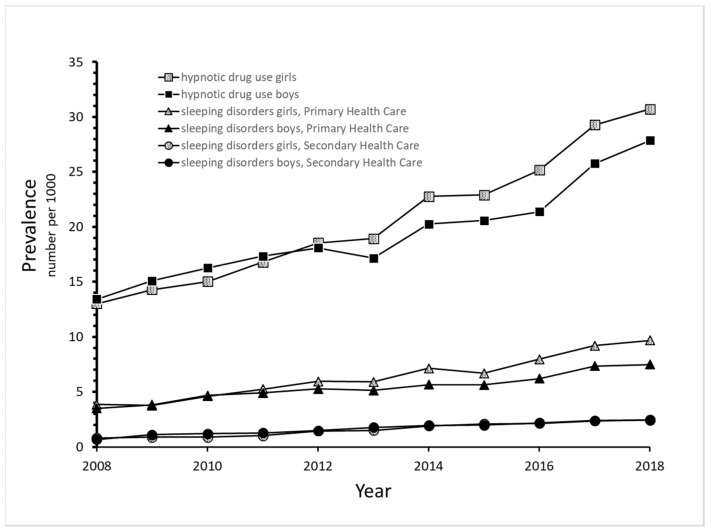
Prevalence of hypnotic drug use during 2008–2018 and sleep disorders diagnosed in primary and secondary health care among adolescents 13–17 years of age.

**Figure 2:**
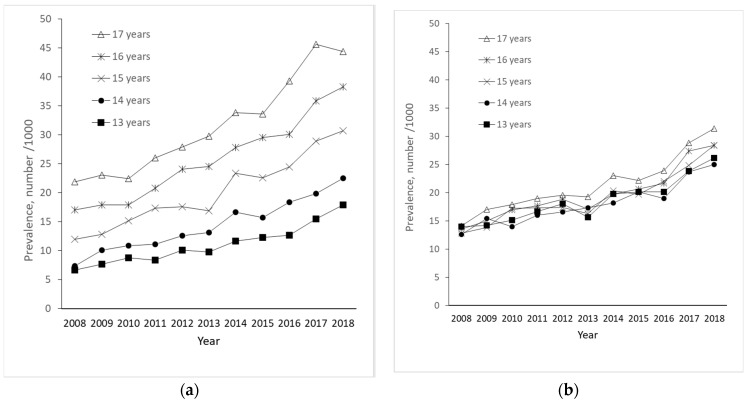
Prevalence of hypnotic drug use during 2008–2018 according to age in girls (**a**) and boys (**b**).

Overall, there was a greater-than-twofold increase in hypnotic drug use from 2008 (N = 4189; 13.2/1000) to 2018 (N = 9150; 29.2/1000), most pronounced among girls (Figure 1). Hypnotic drug use increased during the study period for all ages in both boys and girls (Figure 2a,b). Among the two youngest age groups, the prevalence of hypnotic drug use was higher in boys than in girls throughout the period. However, although the prevalence of use was similar across ages throughout the period in boys, a marked age variation with a higher level of use by older girls was observed.

### 3.2. Annual Prevalence of Sleep Disorder Diagnosis in Primary and Secondary Health Care

The number and prevalence of adolescents who were diagnosed with sleep disorders in primary health care increased by more than twofold in girls and boys during the study period, from 591 (3.8/1000) in 2008 to 1475 (9.7/1000) in 2018 for girls and from 572 (3.5/1000) to 1198 (7.5/1000) for boys (Figure 1). 

In secondary health care, the prevalence of sleep disorders was low but increased from 0.7 and 0.8 per 1000 in 2006 to 2.5 and 2.4 per 1000 in 2018 for boys and girls, respectively (Figure 1).

### 3.3. Incident Hypnotic Drug Users

(a) Incidence rate

**Figure 3:**
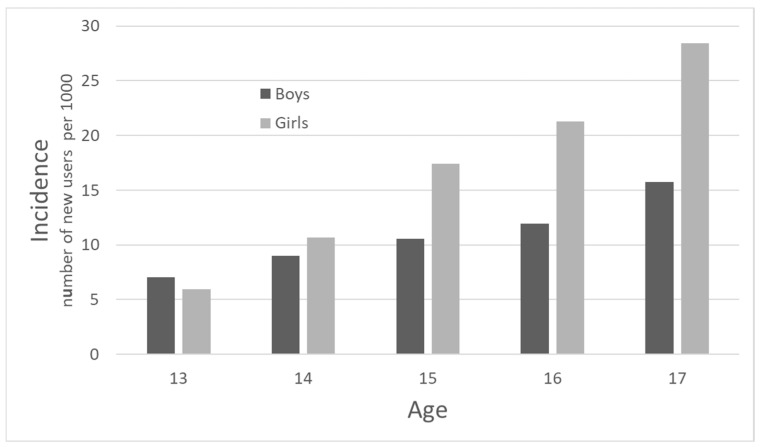
Incidence of hypnotic drug use among adolescents according to age in 2017.

In 2017, there were 2519 girls (16.8 per 1000) and 1718 boys (10.9 per 1000) who were incident users. Incidence was four times greater among 17-year-old girls (28.4/1000) compared to 13-year-olds, and twice as high among 17-year-old boys (15.7/1000) compared to 13-year-olds (Figure 3).

(b) Type of first hypnotic drug use and amount dispensed to incident hypnotic drug users

Melatonin was the first hypnotic drug prescribed for almost 70% of all incident users in 2017, with no gender difference, followed by alimemazine, which accounted for about 27% of incident use in both boys and girls. Low proportions of adolescents were prescribed zopiclone, zolpidem, nitrazepam, or flunitrazepam as their first hypnotic drug (Table 1).

The median number of prescriptions filled during the 365-day period after and including the first filled hypnotic drug prescription was two, and the median amount of drug dispensed in this period was 90 DDDs in both genders (Table 2). The youngest boys and girls were dispensed the highest amount of hypnotic drug during their first year of treatment, 180 and 150 DDD/year, respectively, and the amount fell with increasing age.

(c) Contact with secondary health care among incident hypnotic drug users

Among incident hypnotic drug users, 82% of girls and 77% of boys were recorded as having had at least one contact with secondary health care during the six-month period before and after the first hypnotic drug prescription in 2017. In the general population of 13–17-year-olds, the one-year prevalence of contact with secondary health care in 2017 was 31% for girls and 30% for boys.

(d) Diagnoses from secondary health care among incident hypnotic drug users

**Figure 4:**
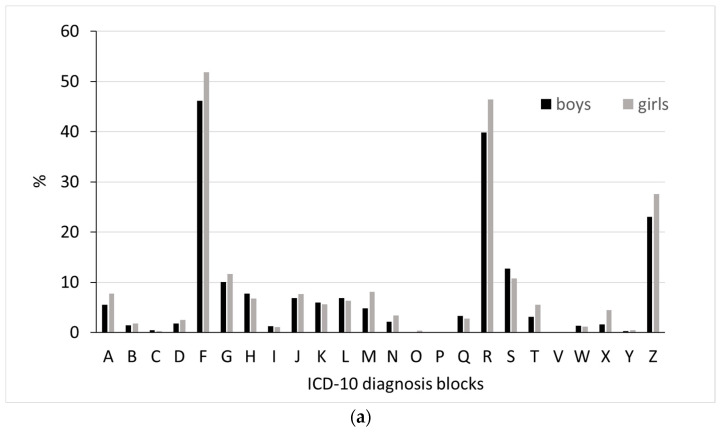

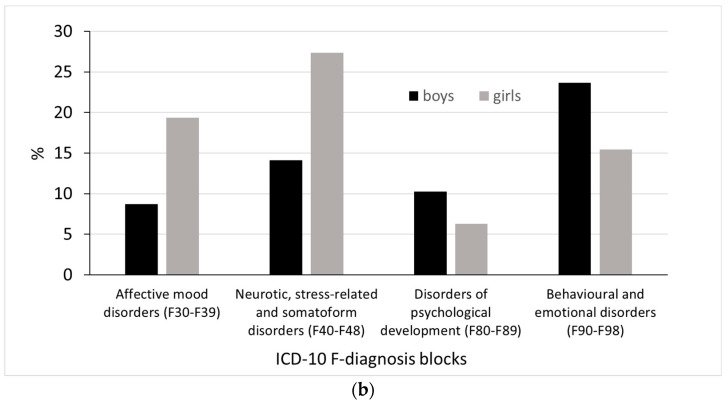
Proportion of (**a**) diagnoses in all ICD-10 diagnosis blocks. A, B, C etc. include A00-A99, B00-B99, C00-C97 etc. And (**b**) the proportion of the most frequent ICD-10 F-diagnoses during a period from six months before to six months after filling the first hypnotic drug prescription among incident adolescent hypnotic users in 2017.

Only 3.2% of incident users had received a diagnosis of a sleep disorder in secondary health care around the time when the first hypnotic drug prescription was dispensed. However, other diagnoses were frequent, mainly represented by the following three ICD-10 diagnoses blocks: F: “Mental, behavioral, and neurodevelopmental disorders” (girls: 51.8%, boys: 46.2%); R: “Symptoms, signs and abnormal clinical and laboratory findings not elsewhere classified” (girls: 46.4%, boys: 39.8%); and Z: “Factors influencing health status and contact with health services” (girls: 27.6%, boys: 23.1%) (Figure 4a). Figure 4b shows the most frequent diagnosis blocks within ICD-10 chapter F for incident hypnotic drug users. “Neurotic stress-related disorders” (F40–48) were most frequent among girls (27.4%), while “Behavioral and emotional disorders” were most common among boys (F90–98) (23.6%) (Figure 4b). 

When focusing on gender differences in more detail, affective mood (F30–39) and neurotic stress-related disorders (F40–48) were more commonly recorded among girls compared to boys, while disorders of psychological development (F80–89) and behavioral and emotional disorders (F90–98) were more commonly recorded among boys compared to girls (Figure 4b).

Within block R, the most recorded diagnosis was R45: Symptoms and signs involving emotional state (girls: 32.3%, boys: 25.3%). Likewise, the most recorded diagnoses in block Z were Z03: Medical observation and evaluation for suspected diseases and conditions ruled out, followed by Z00: General examination and investigation of persons without complaint and reported diagnosis. The proportions receiving these diagnoses were similar for girls and boys, 7.8 and 6.7%, respectively.

(e) Diagnoses during Childhood among incident hypnotic drug users

Incident hypnotic drug users were more commonly recorded as having received an ICD-10 diagnosis from Secondary Health Care during childhood (up to 12 years of age) than the general population for almost all diagnosis blocks (Table 3). This was most pronounced for infections (ICD-10-block A) and mental and behavioral disorders (ICD-10-block F), which were recorded almost five and three times more frequently among incident hypnotic drug users compared to the general population.

## 4. Discussion

The current study provides new insight into the prescription of hypnotic drugs and possible underlying disorders in a nationwide study population of 13–17-year-old adolescents. There has been an increase in both hypnotic drug use and diagnosed sleep disorders among Norwegian adolescents over the last decade, most noticeably among girls. However, the prevalence of hypnotic drug use was markedly higher than the prevalence of diagnosed sleep disorders, implying that hypnotic drugs were largely prescribed to adolescents with other disorders than primary sleep disorders. Incident hypnotic drug users had approximately 2.5 times more contact with secondary health care around the time of the initiation of hypnotic drug use compared to adolescents in the general population. These users seemed to have a high burden of disease, mainly of psychiatric disorders. Among girls, “Neurotic stress-related disorders” were the most frequent, while “Behavioral and emotional disorders” were the most common in boys. Incident hypnotic drug users also had a higher frequency of psychiatric and somatic disease during childhood compared to the general population of adolescents. 

Our results are in accordance with data from other Nordic countries showing an increased use of hypnotic drugs over the last two decades [2,4,8,9]. A recent review and meta-analysis, mostly including studies from Europe and North America, found that systematic monitoring of psychotropic drug use was lacking, results were heterogeneous, and data on hypnotics was limited [1]. 

The prevalence of hypnotic drug use and diagnosis of sleep disorders in primary and secondary health care showed a parallel trend of a threefold increase during the study period but at very different levels. The increase in dispensed drugs and diagnoses for sleep disorders is consistent with the observed increase in self-reported sleep onset difficulties in Norwegian adolescents over the last few decades [13]. Self-reported sleep problems in adolescents seem to lie between 17% [13] and 20–30% [12,23], which is much higher than the recorded prevalence of diagnosed sleep disorders in our study. A general increase in psychological problems is also evident from self-reporting in surveys among Norwegian adolescents [15,24,25], and this may be associated with the increased use of hypnotic drugs observed in our study. A recent ecological study by Lien et al. also showed a significant upward trend in self-reported depressive symptoms and hypnotic drug use over recent decades following the introduction of social media platforms in Norway [26]. 

Most adolescents initiated on hypnotic drugs had other diagnoses than primary sleep disorders in secondary health care. Primary sleep disorders are more frequently diagnosed in primary health care, while adolescents with psychiatric disorders are usually referred to secondary health care. Sleep problems are a symptom of several psychiatric disorders, which could be the reason for prescribing hypnotic drugs to adolescents who are not diagnosed with a primary sleep disorder. 

The results also show that incident hypnotic drug users were in much more contact with the health care system than the general population around the first time their hypnotic drug was dispensed. Incident users also seemed to have had a high disease burden during childhood since they had a higher prevalence of most diagnosis blocks compared to adolescents in the general population. In line with our results, a high disease burden was also found among Swedish adolescents, where half of melatonin users had at least one psychiatric diagnosis around the time of prescription [9]. The results also concur with previous investigations showing a high degree of comorbidity among 4–17-year-old recurrent users of melatonin [7], a high degree of comedication in 0–17-year-old users of hypnotic drugs [6], and a substantial proportion of concurrent psychopathologies among 0–24-year-old users [11]. Thus, the finding that incident users of hypnotic drugs had such a high disease burden requiring specialist health care services emphasizes how adolescents who are initiated on hypnotic drugs do have severe underlying morbidity. The increase in the use of hypnotic drugs needs to be discussed in this context. 

Both the incidence and prevalence of hypnotic drug use were higher in boys than girls in the youngest age groups (13- and 14-year-olds). Nevertheless, during adolescence, girls showed a higher increase in hypnotic drug use compared to boys, reaching a prevalence of nearly 5% among 17-year-old girls compared to almost 3% among 17-year-old boys in 2017. This concurs with previous data showing marked gender differences in psychopathology during childhood and adolescence, with male preponderance in early-onset disorders and female preponderance in adolescent-onset disorders [27]

Neurotic stress-related disorders and affective mood disorders, including anxiety disorders and depression, were the most prevalent disorders recorded among female incident hypnotic drug users in our study. In contrast, behavioral disorders, including hyperkinetic disorder, were the most frequently diagnosed disorder in male hypnotic drug users. During adolescence, many psychiatric disorders emerge [28], and there is evidence of gender differences in adolescents and adults developing different psychiatric and mental problems [29,30]. The gender variation in the diagnosis profiles of hypnotic drug users can be seen in context with our observation of high prevalence of use in 13-year-old boys. Hyperkinetic disorder is often diagnosed at an earlier age and is more common in boys than in girls [31]. Boys with hyperkinetic disorder may already have started melatonin use before the age of 13 years. A Swedish report [9] also supports the assumption of gender differences in diagnosis by showing that 13–17-year-old girls are diagnosed with depression almost three times more frequently than boys. The observed patterns of hypnotic drug use in girls and boys may reflect patterns of psychiatric and neurodevelopmental morbidities and their time of occurrence in girls and boys.

The most frequently prescribed hypnotic drug in incident users was melatonin, followed by alimemazine, with no gender difference in the choice of drug. Melatonin has for several years been the most frequently used hypnotic drug among Norwegian children and adolescents, accounting for a major part of the increase in the use of hypnotics in this population [6,7]. A 15–20-fold increase in melatonin use was also observed in Sweden in children aged between 0 and 17 years from 2006 to 2017, confirming a similar trend to that in Norway [9]. An increase in melatonin use among children and adolescents has also been reported in Denmark [8]. A recent Scandinavian study also showed a continued increase in the use of melatonin and sedative antihistamines in young people during 2012–2018 [2].

Melatonin’s effect on insomnia is best documented in children and adolescents with neurodevelopmental disorders [32,33,34]. Melatonin has only recently been approved for the treatment of insomnia in children and adolescents with ASD and ADHD. The results of the present study showed that most children had diagnoses other than the two approved ones, particularly girls. This indicates that off-label use of prescribed melatonin is still common.

The annual amount of drugs dispensed during the first year after initiation of hypnotic drug treatment indicates that their use was more than just sporadic. The median number of DDDs dispensed covers several months of daily use when assuming an intake of one DDD per day. The median number of two prescriptions filled also indicates more regular use. In both genders, the highest annual dose was seen in the youngest age group (13 years). In particular, 13-year-old boys seemed to be more regular users once they started. Around 70% of hypnotic drug users were initiated on melatonin, and melatonin was the major contributor to the overall annual amount used in terms of DDDs. The short-term use of melatonin is believed to be safe, even in high dosages, in adults. However, the safety of long-term use has not been clarified in adolescents, and caution is required, particularly in relation to puberty and reproduction [35].

Alimemazine was the second most common hypnotic drug, initiated in around one out of four new adolescent users. A recently published Scandinavian study [2] revealed that males are twice as likely to use melatonin before the age of 15 compared to females, and that females are more likely to use z-drugs and sedative antihistamines after the age of 15 years compared to males. Furthermore, the estimated quantity of use throughout a year indicated regular use of melatonin and a more sporadic/short-term use of sedative antihistamines and z-drugs. For several years in Norway, alimemazine has been the dominating sleep-inducing drug in toddlers, but use has declined [5]. Its use has generally been much lower in adolescents than in toddlers [36]. There is limited evidence of both the effect and safety of alimemazine in children and adolescents [37]. However, it seems that alimemazine is the preferred hypnotic drug over other potentially addictive drugs in many adolescents with a disease burden in need of specialist health care. Even though we observed an increase in hypnotic drug use in Norway, the potentially addictive z-hypnotics (zopiclone and zolpidem) and benzodiazepines (nitrazepam and flunitrazepam) were rarely used in the adolescent population, in accordance with the recommendations.

It must be noted that we did not investigate the diagnosing doctors’ specialties. It might be that the medical specialties of the diagnosing doctors could explain why both “Mental, Behavioral and Neurodevelopmental disorders” (ICD-10 block F) and “Symptoms and signs involving emotional state” (block R) were the most prevalent diagnoses. Specialists in psychiatry may be more familiar with diagnoses in ICD-10 block F, whereas non-psychiatrists may prefer to use the more unspecific symptom diagnoses in block R. A Swedish report showed that melatonin was most frequently prescribed by specialists in psychiatry (more than 50%), and to a lesser degree by specialists in pediatrics/internal medicine or rehabilitation [9]. 

A major strength of the present study is the use of data from national registries, including the entire Norwegian population, which minimizes selection bias and recall bias. Another strength is the linkage of data from the NorPD and NPR on an individual level. These results are not influenced by the over-the-counter sale of melatonin because data were collected from the period when melatonin was only available on prescription.

One limitation of the present study is that we have no information about drugs administered to hospitalized adolescents. However, in Norway, few adolescents are hospitalized for long periods, so we do not expect this lack of information to substantially influence our results, in particular not time-trends. In addition, dispensed drugs registered in the NorPD do not give information about actual drug intake. Drug use defined by dispensed drugs as recorded in the NorPD will include both non-users, sporadic users, and more regular users. We did not have the possibility to link individual-level data on diagnoses from primary health care (KUHR) to prescription data and could, therefore, only study diagnoses from secondary health care in incident hypnotic drug users.

The increasing use of hypnotic drugs among adolescents indicates a need for more treatments for sleeping problems. It is important that non-pharmacological approaches are also considered. Cognitive behavioral therapy (CBT) for insomnia is the first-line treatment for adults, and existing studies also show promising effects for children and adolescents [38]. CBT is not optimally implemented in clinical settings since the demand for face-to-face CBT exceeds supply [39]. Digital CBT has been shown to be a moderately effective treatment for insomnia for children and may contribute to an increased use of CBT in young patients in the years to come [40].

The trend of increasing use of hypnotic drugs in adolescents mainly reflects the initiation of hypnotic drugs in a subgroup of the population with a higher disease burden, mainly of psychiatric disorders, than the general population. Adolescents were primarily prescribed melatonin. Even though melatonin has a milder side effect profile than, for example, benzodiazepines or z-hypnotics, its long-term effect and safety need to be investigated, especially among children and adolescents with psychiatric comorbidities. 

## Figures and Tables

**Table 1:** Percent distribution of the first hypnotic drug filled by incident ^†^ users in 2017.

Hypnotic Drug	Boys		Girls	
	n	%	n	%
melatonin	1168	68.0	1738.0	69.0
alimemazine	465	27.1	675	26.8
zopiclone	58	3.4	71	2.8
zolpidem	16	0.9	22	0.9
nitrazepam	<11	-	<13	-
flunitrazepam	<5	-	<5	-

^†^ Incident use is defined as no hypnotic drug dispensed in the 2 years before the first dispensed hypnotic drug in 2017. All hypnotic drug types dispensed are included.

**Table 2:** Amount and number of filled prescriptions of hypnotic drugs used by incident ^†^ users (13–17 years) in the 365 days after the first filled hypnotic drug prescription in 2017.

Age (Years)	DDD/365 Days		Number of Prescriptions Filled/365 Days	
	Median (IQR)		Median (IQR)	
	Girls	Boys	Girls	Boys
13	150 (56–360)	180 (90–450)	2.5 (1–5)	3 (1–5)
14	120 (59–360)	120 (78–360)	2 (1–4)	2 (1–4)
15	90 (30–270)	98 (30–270)	2 (1–4)	2 (1–4)
16	90 (30–194)	90 (30–227)	2 (1–3)	2 (1–4)
17	90 (30–180)	69 (30–150)	2 (1–3)	1 (1–3)

^†^ Incident use is defined as no hypnotic drug dispensed in the 2 years before the first hypnotic drug was dispensed in 2017. DDD—defined daily dose; IQR—interquartile range.

**Table 3:** Number and proportion of boys and girls aged 13–17 years in 2017 in the general population and among incident ^†^ hypnotic drug users with diagnosis from specialist health care before the age of 12 in ICD-10 blocks.

ICD-10 Diagnosis Blocks	Boys					Girls				
	General Population		Hypnotic Users			General Population		Hypnotic Users		
	n = 189,900		n = 1718		*p*	n = 179,270		n = 2519		*p*
	n	%	n	%		n	%	n	%	
A	2914	1.6	132	7.7	<0.001	2465	1.4	190	7.5	<0.001
B	4747	2.6	62	3.6	<0.001	5278	2.9	97	3.9	<0.001
C	391	0.2	8	0.5	0.019	302	0.2	7	0.3	0.186
D	4922	2.7	48	2.8	0.600	4952	2.8	77	3.1	0.371
E	5891	3.3	0	0.0	<0.001	6568	3.7	0	0.0	0.001
F	22,960	12.8	622	36.2	<0.001	12,417	6.9	492	19.5	<0.001
G	7349	4.1	161	9.4	<0.001	5670	3.2	161	6.4	<0.001
H	30,726	17.1	362	21.1	<0.001	28,916	16.1	552	21.9	<0.001
I	2109	1.2	31	1.8	0.006	1789	1.0	34	1.3	0.078
J	26,860	15	316	18.4	<0.001	20,920	11.7	396	15.7	<0.001
K	13,903	7.8	218	12.7	<0.001	12,493	7.0	315	12.5	<0.001
L	10,550	5.9	119	6.9	0.014	10,884	6.1	206	8.2	<0.001
M	10,563	5.9	166	9.7	<0.001	10,650	5.9	244	9.7	<0.001
N	10,142	5.7	147	8.6	<0.001	4440	2.5	99	3.9	<0.001
O	<5		<5		*	25	0.0	<5		*
P	269	0.2	<5		*	155	0.1	6	0.2	0.011
Q	11,691	6.5	145	8.4	<0.001	8654	4.8	160	6.4	<0.001
R	28,777	16.1	463	26.9	<0.001	24,397	13.6	616	24.5	<0.001
S	52,897	29.5	605	35.2	<0.001	46,038	25.7	968	38.4	<0.001
T	9842	5.5	127	7.4	<0.001	7668	4.3	148	5.9	<0.001
U	9	0.0	0	0.0	0.775	19	0.0	0	0.0	0.605
V	947	0.5	5	0.3	0.223	614	0.3	17	0.7	0.005
W	5874	3.3	82	4.8	<0.001	4972	2.8	108	4.3	<0.001
X	1147	0.6	24	1.4	<0.001	889	0.5	22	0.9	0.008
Y	235	0.1	<5		*	167	0.1	<5		*
Z	58,461	32.6	887	51.6	<0.001	48,874	27.3	1055	41.9	<0.001

^†^ Incident use is defined as no hypnotic drug dispensed in the 2 years before the first dispensing in 2017. * <5 denotes fewer than five individuals in the group. Exact numbers are not shown due to privacy protection regulations and therefore no *p* values are shown.

## Data Availability

The datasets generated and analyzed during the current study are not publicly available due to the restrictions imposed by Norwegian legislation for privacy protection.

## Supplementary Materials

The following supporting information can be downloaded at: https://www.mdpi.com/article/10.3390/jcm13041075/s1, Table S1: ATC codes and DDDs for the hypnotic drugs used by the study population.; Table S2: ICD-10 chapters, WHO version.
