# Supplementary material for: Morbidity among Adolescent Hypnotic Drug Users in Norway: An Observational Population-Based Study
Source: J Clin Med. 2024 Feb 14;13(4):1075. doi: 10.3390/jcm13041075 (PMC10888536; doi:10.3390/jcm13041075)
Supplement: Supplementary file 1 [file jcm-13-01075-s001.zip › jcm-2812098-supplementary.pdf]

Table S1

ATC codes and DDDs for the hypnotic drugs used by the study population

**Drug**	**ATC code**	**1 DDD (oral adm.)**
nitrazepam	N05CD02	5 mg
flunitrazepam	N05CD03	1 mg
zopiclone	N05CF01	7.5 mg
zolpidem	N05CF02	10 mg
melatonin	N05CH01	2 mg
alimemazine	R06AD01	30 mg

ATC - Anatomical Therapeutic Chemical

DDD - Defined Daily Dose

Table S2

ICD-10 chapters, WHO version

Chapter	Block	Description
I	A00-B99	Certain infectious and parasitic diseases
II	C00-D48	Neoplasms
III	D50-D89	Diseases of the blood and blood-forming organs and certain disorders involving the immune mechanism
IV	E00-E90	Endocrine, nutritional and metabolic diseases
V	F00-F99	Mental and behavioral disorders
VI	G00-G99	Diseases of the nervous system
VII	H00-H59	Diseases of the eye and adnexa
VIII	H60-H95	Diseases of the ear and mastoid process
IX	I00-I99	Diseases of the circulatory system
X	J00-J99	Diseases of the respiratory system
XI	K00-K93	Diseases of the digestive system
XII	L00-L99	Diseases of the skin and subcutaneous tissue
XIII	M00-M99	Diseases of the musculoskeletal system and connective tissue
XIV	N00-N99	Diseases of the genitourinary system
XV	O00-O99	Pregnancy, childbirth and the puerperium
XVI	P00-P96	Certain conditions originating in the perinatal period
XVII	Q00-Q99	Congenital malformations, deformations and chromosomal abnormalities
XVIII	R00-R99	Symptoms, signs and abnormal clinical and laboratory findings, not elsewhere classified
XIX	S00-T98	Injury, poisoning and certain other consequences of external causes
XX	V01-Y98	External causes of morbidity and mortality
XXI	Z00-Z99	Factors influencing health status and contact with health services
XXII	U00-U85	Codes for special purposes
